# In this issue – Food insecurity

**DOI:** 10.1017/S136898002200060X

**Published:** 2022-04

**Authors:** Allison Hodge

**Affiliations:** Cancer Council Victoria, Victoria, Australia

The second of the Sustainable Development Goals (SDG) is ‘End hunger, achieve food security and improved nutrition and promote sustainable agriculture’. Target 2.1.2 used to assess achievement of the goals is ‘By 2030, end hunger and ensure access by all people, in particular the poor and people in vulnerable situations, including infants, to safe, nutritious and sufficient food all year round’. The 2021 report on the status of the SDG notes that the current status of this goal is ‘very far from target’ and the trend is ‘deterioration’^(1)^. The importance and prevalence of food insecurity globally is evidenced by the articles selected in this issue of *Public Health Nutrition*.

Of twenty-eight articles in this issue, fourteen addressed food security/insecurity in different contexts.

Among more than 3 million US Veterans who have been screened for food insecurity since 2017, 1·3 % of men and 2·0 % of women were food insecure. This was particularly associated with medical and trauma-related co-morbidities^(2)^.

In Finland, food insecurity was assessed among 6435 private sector service workers recruited through their union. Sixty-five per cent of respondents were food insecure and 36 % severely so. Young age and difficulty covering household expenses were strong risk factors. Even in these employed people in a high-income country, food insecurity was a common occurrence^(3)^.

In a study from Botswana, 180 people newly diagnosed with tuberculosis, about half of whom were HIV-positive, were studied, and food insecurity was found to be associated with increased risk of depression and anxiety irrespective of HIV status^(4)^.

Using data from almost 3000 adolescents (aged 12–17 years), in low-income households from the National Health and Nutrition Examination Survey (2007–2016), household and individual food insecurities were studied in relation to cardiovascular risk factors. Overall, 33 % of the adolescents experienced household food insecurity and 17 % individual food insecurity using population weighting. In contrast with findings in adults, there was no association between food insecurity and risk factors (BMI, blood pressure, lipids and glucose) in this sample^(5)^.

Between 2013 and 2020, people in four African countries were surveyed to assess their level of food insecurity. Approximately half of the 3500 participants were food insecure according to their definition, and this did not vary by HIV status. Older age and more dependents were associated with more food insecurity, while better education and antiviral therapy in those living with HIV were associated with lower risk of food insecurity^(6)^.

Four separate studies on food insecurity were conducted in Bage in Brazil during May–June 2020 during the COVID-19 pandemic. The prevalence of household food insecurity was 29 %, and it was associated with the occurrence of major depressive episodes (MDE) irrespective of the criteria used to define major depressive episodes based on data from the Patient Health Questionnaire^(7)^.

A study of health system members in Detroit, USA, found that geographic proximity to healthy grocery stores or access to a car was not associated with food insecurity, but some areas were at increased risk of food insecurity that had an overall prevalence of 31 %^(8)^.

A more in-depth study of thirty people in Belize identified family composition, income, education and employment as factors that influenced individuals’ ability to afford and access food for themselves or their families. Transportation and distance to food sources also posed challenges to accessing suitable food^(9)^.

Another article describes a systematic review and meta-analysis looking at food insecurity in urban households in east African countries. Seventeen studies with data from eight countries were included with 61 % of households being food insecure, ranging from 36 % in Burundi to 91 % in Sudan. Limited education, female household head, larger family size and low wealth were associated with food insecurity^(10)^.

In Iran, household food insecurity was assessed in 2500 women during the third trimester of pregnancy and infant anthropometric measures collected during the first 6 months of life. Over 30 % or participants experienced some degree of food insecurity and children born into these households were more likely to be stunted at birth, 4 months and 6 months^(11)^.

Data from the 2015 Canadian Community Health Survey-Nutrition was used to assess the association between food security and diet quality in children and adults. An important limitation is the use of a single 24-h recall to represent diet. Several variables were considered to represent diet quality, and the proportion of energy from ultra-processed foods was most strongly associated, with higher intake in the least food secure households across all ages^(12)^.

It is understood that the COVID-19 pandemic increased food insecurity. This study in the USA interviewed people who were clients of a food pantry and enrolled in the Supplemental Nutrition Assistance Program before the pandemic using in-depth phone interviews to evaluate the impact of COVID-19 on food insecurity and associated outcomes. The study found that (i) the pandemic increased economic distress and (ii) the pandemic increased food needs, food prices and food shortages. In combination with economic stressors, this led to greater food insecurity; (iii) increased economic stress and food insecurity contributed to increased psychological stress, associated with fear of infection, isolation and children being confined at home^(13)^. In a study from North Carolina, USA, food insecurity was associated with infant mortality rate at a county level^(14)^.

The studies noted above have highlighted the prevalence of food insecurity, albeit assessed in different ways, some of the risk factors associated with it and some potential adverse consequences. The last study has attempted to assess the effects of the PROSPERA programme on food insecurity in Mexico between 2012 and 2016. PROSPERA was a conditional cash transfer programme that allocated cash, conditional on children being enrolled in school, towards achieving nutritional gains in beneficiary households, including eliminating acute child malnutrition and improving child weight and height indicators. The programme appears to have reduced food insecurity in recipient households, and similar programmes have been launched in many countries^(15)^. There are likely to be many ways to address food insecurity in the long and short term and many benefits. I look forward to reading more papers identifying and describing such successes.

## References

[1] FaAOotU Nations (2021) Tracking Progress on Food and Agriculture-Related SDG Indicators 2021. Food and Agriculture Orgainzation of the United Nations.

[2] Cohen AJ , Dosa DM , Rudolph JL et al. (2021) Risk factors for Veteran food insecurity: findings from a National US Department of Veterans Affairs Food Insecurity Screener. Public Health Nutr. Published online: 9 November 2021. 10.1017/S1368980021004584 PMC8957505 34743780

[3] Walsh HM , Nevalainen J , Saari T et al. (2022) Food insecurity among Finnish private service sector workers: validity, prevalence and determinants. Public Health Nutr. Published online: 25 January 2022. 10.1017/S1368980022000209 PMC9993037 35067259

[4] Wang Q , Dima M , Ho-Foster A et al. (2020) The association of household food insecurity and HIV infection with common mental disorders among newly diagnosed tuberculosis patients in Botswana. Public Health Nutr. Published online: 20 October 2020. 10.1017/S1368980020004139 PMC8053731 33070794

[5] Fulay AP , Vercammen KA , Moran AJ et al. (2021) Household and child food insecurity and CVD risk factors in lower-income adolescents aged 12–17 years from the National Health and Nutrition Examination Survey (NHANES) 2007–2016. Public Health Nutr. Published online: 23 June 2021. 10.1017/S1368980021002652 PMC9991548 34155968

[6] Onyenakie CC , Nnakwe RU , Dear N et al. (2021) Prevalence and predictors of food insecurity among people living with and without HIV in the African Cohort Study. Public Health Nutr. Published online: 24 August 2021. 10.1017/S136898002100361X PMC9991625 34420547

[7] Santos LP , Schafer AA , Meller FO et al. (2021) Association between food insecurity and major depressive episodes amid Covid-19 pandemic: results of four consecutive epidemiological surveys from southern Brazil. Public Health Nutr. Published online: 25 November 2021. 10.1017/S1368980021004626 PMC9991800 34814966

[8] Santarossa S , Hill AB , Sitarik AR et al. (2021) Food insecurity in Detroit: exploring the relationship between patient-reported food insecurity and proximity to healthful grocery stores. Public Health Nutr. Published online: 31 July 2021. 10.1017/S1368980021003128 PMC9991681 34325766

[9] Stevenson LD , Reznar MM , Onye E et al. (2021) A qualitative inquiry of food insecurity in Belize. Public Health Nutr. Published online: 12 June 2021. 10.1017/S1368980021002615 PMC9991813 34114538

[10] Gebremichael B , Beletew B , Bimerew M et al. (2021) Magnitude of urban household food insecurity in East Africa: a systematic review and meta-analysis. Public Health Nutr. Published online: 17 August 2021. 10.1017/S1368980021003529 PMC9991803 34392860

[11] Karbin K , Khorramrouz F , Farkhani EM et al. (2021) Household food insecurity during pregnancy as a predictor of anthropometric indices failures in infants aged less than 6 months: a retrospective longitudinal study. Public Health Nutr. Published online: 21 August 2021. 10.1017/S1368980021003591 PMC9991822 34412726

[12] Hutchinson J & Tarasuk V. (2021) The relationship between diet quality and the severity of household food insecurity in Canada. Public Health Nutr. 2021. Published online: 24 September 2021. 10.1017/S1368980021004031 PMC9991759 34551845

[13] Higashi RT , Sood A , Conrado AB et al. (2021) Experiences of increased food insecurity, economic and psychological distress during the COVID-19 pandemic among Supplemental Nutrition Assistance Program-enrolled food pantry clients. Public Health Nutr. Published online: 12 July 2021. 10.1017/S1368980021004717 PMC8712963 34865672

[14] Cassidy-Vu L , Way V , & Spangler J. (2022) The correlation between food insecurity and infant mortality in North Carolina. Public Health Nutr. Published online: 1 February 2022. 10.1017/S136898002200026X PMC9991794 35094744

[15] Saldivar-Frausto M , Unar-Munguia M , Mendez-Gomez-Humaran I et al. (2021) Effect of a conditional cash transference program on food insecurity in Mexican households: 2012–2016. Public Health Nutr. Published online: 10 September 2021. 10.1017/S1368980021003918 PMC9991821 34497003

